# Un myofibroblastome de type mammaire pas comme les autres: à propos d’un cas

**DOI:** 10.11604/pamj.2017.26.69.11339

**Published:** 2017-02-20

**Authors:** Othmane Lahbali, Amine Azami, Mohammed Tbouda, Adil Elyamine, Fouad Zouaidia, Najat Mahassini

**Affiliations:** 1 Service d’Anatomie Pathologique, Centre Hospitalier Universitaire Ibn Sina, Rabat, Maroc; 2 Service d’Histologie, Centre Hospitalier Universitaire Ibn Sina, Rabat, Faculté de Médecine de Casablanca, Maroc; 3 Laboratoire d’Anatomie Pathologique Centre Hospitalier Universitaire Ibn Sina Rabat, Maroc

**Keywords:** Myofibroblastome de type mammaire, diagnostic différentiel, Maroc, Mammary-type myofibroblastoma, differential diagnosis, Morocco

## Abstract

Le myofibroblastome de type mammaire (MM) est une tumeur mésenchymateuse bénigne rare initialement décrit dans le sein. Le diagnostic de cette entité repose sur un spectre d’arguments cliniques radiologiques et éventuellement histologiques. Nous rapportons un cas rare de myofibroblastome de type mammaire siégeant au niveau axillaire chez un homme de 50 ans. La particularité de notre cas est l’existence de plusieurs figures de mitoses et ceci est rarement décrit dans la littérature. A travers ce cas nous soulevons les problématiques du diagnostic différentiel de cette tumeur rare.

## Abstract (en)

Mammary-type myofibroblastoma (MTMF) is a rare benign mesenchymal tumor, initially detected in the breast. Its diagnosis is based on a spectrum of clinical, radiological and possibly histological parameters. We here report the rare case of a 50 year old male patient with mammary-type myofibroblastoma located in the axillary region. The peculiarity of our case lies in the presence of numerous mitoses and this was rarely described in the literature. The aim of our case study was to raise the problem of differential diagnosis of this rare tumor.

## Introduction

Le myofibroblastome de type mammaire est une tumeur mésenchymateuse bénigne rare initialement décrit par Wartoz au niveau mammaire. Cette entité peut siéger dans d’autres sites extra-mammaire, nous rapportons un cas rare de myofiroblatstome de type mammaire au niveau axillaire, la particularité de notre cas est l’aspect morphologique inhabituelle [1]. A travers ce cas nous exposant les principaux diagnostics différentiels de cette entité.

## Patient et observation

**Figure 1:**
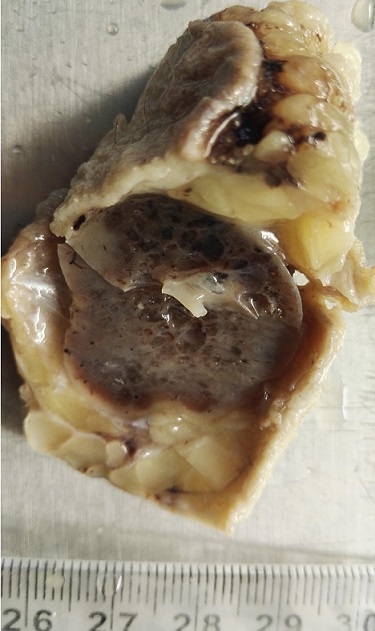
Lésion nodulaire blanchâtre blanc grisâtre bien limitée

**Figure 2:**
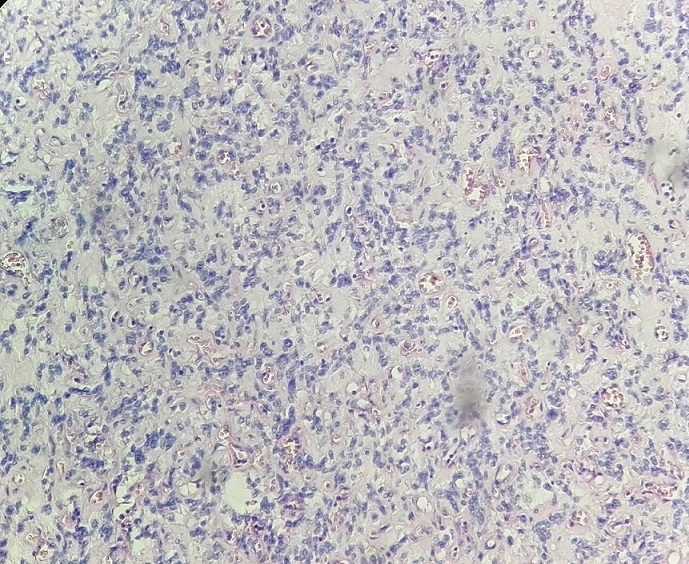
Prolifération agencée en faisceaux courts séparés par des trousseaux de collagène

**Figure 3:**
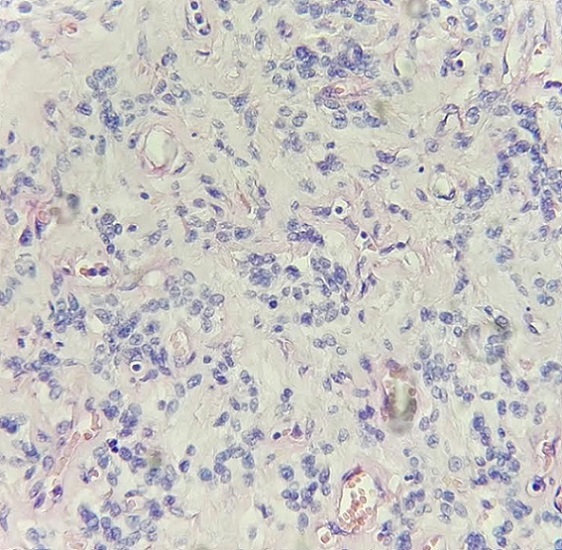
Cellules fusiformes et rondes séparées par des trousseaux de collagène

Nous rapportons le cas d’un homme âgé de 50 ans qui présentait depuis 2 ans une lésion nodulaire bien circonscrite au niveau axillaire gauche mobile par rapport au plan profond et superficiel, la peau en regard était normale. Il n’a pas été individualisé à l’examen des aires ganglionnaires d’adénopathie. L’examen des seins était sans anomalie. Le reste de l’examen clinique était sans particularité. Sur le plan radiologique la masse était bien limitée mesurant 5 cm de grand axe sans calcifications. Une exérèse chirurgicale a été faite objectivant à l’examen macroscopique une masse nodulaire blanc grisâtre mesurant 5 cm de grand axe de consistance ferme (Figure 1). L’examen microscopique de cette lésion montre au niveau dermique une prolifération fusocellulaire agencée en faisceaux courts irréguliers séparés par des trousseaux de collagène hyalinisés d’épaisseur variable (Figure 2). Les cellules sont souvent fusiformes parfois rondes montrant quelques figures de mitoses (Figure 3). La densité cellulaire est variable avec présence d’un infiltrat inflammatoire polymorphe comportant quelques mastocytes. Devant cet aspect indifférencié une étude immunohistochimique a été réalisée montrant un marquage positif des cellules au CD34 confirmant le diagnostic myofibroblastome de type mammaire.

## Discussion

Le myofibroblastome de type mammaire est une tumeur conjonctive bénigne rare à différenciation myofibroblastique, individualisée initialement par Wargotz en 1987 [1]. Cliniquement la plupart des patients présentaient un nodule solitaire bien circonscrit de croissance lente [2], au niveau mammaire les lésions apparaissent le plus souvent chez les femmes ménopausées et chez les hommes plus âgés. Dans l'étude originale de Wargotz et al. [1], l'âge moyen au moment du diagnostic était de 63 ans. Les lésions extra-mammaires apparaissent le plus fréquemment chez les hommes plus âgés, et des cas ont été signalés dans une grande variété de sites anatomiques, y compris les fesses [3], vulve [4], région péri-anale [5], région para-testiculaire [6], les extrémités [7], et au niveau de la tête et du cou. L’étude menée par Brooke E. el al [8], montre que le site le plus fréquent était la région inguinale /périnéale. L'imagerie montre une masse bien délimitée, homogène et solide dépourvue de microcalcifications [2, 9]. La bilatéralité et la multicentricité ont rarement été observées [9]. Macroscopiquement cette tumeur est souvent bien limitée blanc grisâtre avec possibilité de remaniement myxoïde, sa taille varie entre 2 et 11 cm, bien que la plupart ne dépasse pas 5 cm au moment de l’excision [2, 10].

Le myofibroblastome de type mammaire englobe un large spectre morphologique, ressemblant souvent à un lipome de cellule fusiforme [2, 11]. Histologiquement, il s’agit d’une tumeur composée d’une prolifération de cellules fusiformes agencée en courts faisceaux irréguliers dissociées par des trousseaux de collagène hyalinisés [1]. Parfois les cellules sont focalement plus rondes d’aspect “pseudo-épithélial” ou de plus grande taille et plurinucléés. Il faut noter que certains cas peuvent présenter une cellularité élevée, des cellules atypiques, des marges infiltrantes, des modifications myxoïdes ou stromales fibreuses étendues [2] et occasionnellement on peut trouver une métaplasie musculaire lisse, cartilagineuse ou osseuse [1, 12, 13]. Les figures de mitoses sont généralement inférieures à 2 mitoses par 10 champs au fort grandissement. Au sein de la lésion, on retrouve des adipocytes matures, mais il n’y pas de structures épithéliales mammaires ou de nécrose. Les cellules fusiformes sont négatives pour les cytokératines, l’EMA et la Protéine S100. Par contre, ces cellules expriment le CD34, l’actine muscle lisse, la desmine et le CD10. Moins de 10% des myofibroblastome du sein peuvent être RO (récepteur oestrogénique) et RP (récepteurs progestatifs) négatifs. Au niveau extra-mammaire cette entité peut être confondue avec un large éventail de néoplasmes mésenchymateux, en particulier le lipome à cellules fusiformes. Ce dernier, bien qu’il est immuno-réactive au CD10 et le CD34 comme le myofibroblastome de type mammaire, il se localise cependant presque exclusivement au niveau de la partie postérieure du cou et de l’épaule et du haut du dos, ainsi il n’exprime pas la desmine.

Néanmoins, comme l'a souligné Pauwels et al. [14], le chevauchement entre ces deux entités est impressionnant (y compris les résultats cytogénétiques) et dans certains cas, la distinction entre eux est quelque peu arbitraire. De même, le myofibroblastome de type mammaire présente des caractéristiques qui se chevauchent avec celles de l'angiofibrome cellulaire, y compris une prédilection à se développé au niveau inguinal [15, 16]. Cette dernière lésion (également appelée angiomyofibroblastome-like Tumor) [16] est une tumeur bénigne à cellules fusiformes caractérisée par une cellularité variable, des vaisseaux sanguins hyalinisés et une immuno-réactivité généralement négative à la fois pour la desmine et l'actine muscle lisse, bien qu'elle soit souvent positive pour le CD34. Parmi les diagnostiques différentiels aussi du MTM on trouve la tumeur fibreuse solitaire (TFS), le lipome atypique et la fibromateuse desmoïde. Ces tumeurs contrairement au MTM ont un risque important de récidive et dans de rare cas de métastase (notamment la TFS). Le MTM est distingué du lipome atypique par l’absence de lipoblastes, d’adipocyte de taille variable et d’atypie nucléaire significative en plus le lipome atypique présente des anomalies génétiques particulières: chromosomes en anneaux et chromosomes géants dérivés des régions q13-15 du chromosome 12.

Sur le plan immunohistochimique, les noyaux des cellules adipeuses du lipome atypique réagissent avec les anticorps anti-MDM2 et anti-CDK4 car ces deux gènes, portés par le chromosome 12, sont classiquement amplifiés dans le lipome atypique [17]. La fibromatose desmoïde peut être envisagée dans le diagnostic différentiel, en particulier du MTM hyalinisé, mais, contrairement au MTM, la fibromatose desmoïde présente de longs fascicules de cellules fusiformes et montre fréquemment l'expression nucléaire de la b-caténine [18]. Il a été suggéré que le myofibroblastome représente simplement une tumeur fibreuse solitaire (TFS) du sein, cette tumeur est caractérisée par une cellularité variable, une vascularisation hémangiopéricytaire et une surexpression du STAT6 un marqueur spécifique et sensible du TFS [19]. Sur le plan génétique cette entité partage avec le lipome à cellules fusiformes et l’angiofibrome cellulaire les mêmes réarrangements chromosomiques affectant la région 13q [14, 20]. Le degré de chevauchement morphologique entre ces entités, en combinaison avec une génétique partagée et une distribution anatomique légèrement chevauchée, soulève la question si ces tumeurs sont ou non de véritables entités distinctes ou plutôt représentent un seul spectre de tumeurs génétiquement apparentées.

## Conclusion

Indépendamment de l'aspect histologique, et de l'emplacement anatomique, le MTM n'a pratiquement aucun potentiel de récidive ou de métastase, même avec des marges d'excision positives [8].
